# Characterizing temporal trends in populations exposed to aircraft noise around U.S. airports: 1995–2015

**DOI:** 10.1038/s41370-023-00575-5

**Published:** 2023-09-21

**Authors:** Daniel D. Nguyen, Jonathan I. Levy, Chanmin Kim, Kevin J. Lane, Matthew C. Simon, Jaime E. Hart, Eric A. Whitsel, Trang VoPham, Andrew Malwitz, Junenette L. Peters

**Affiliations:** 1 Department of Environmental Health, Boston University School of Public Health, Boston, MA USA; 2 Department of Biostatistics, Boston University School of Public Health, Boston, MA USA; 3 Department of Statistics, Sungkyunkwan University, Seoul, South Korea; 4 John A. Volpe National Transportation Systems Center, U.S. Department of Transportation, Cambridge, MA USA; 5 Channing Division of Network Medicine, Department of Medicine, Brigham and Women’s Hospital and Harvard Medical School, Boston, MA USA; 6 Exposure, Epidemiology and Risk Program, Department of Environmental Health, Harvard T.H. Chan School of Public Health, Boston, MA USA; 7 Department of Epidemiology, Gillings School of Global Public Health, University of North Carolina at Chapel Hill, Chapel Hill, NC USA; 8 Department of Medicine, University of North Carolina at Chapel Hill, Chapel Hill, NC USA; 9 Department of Epidemiology, University of Washington, Seattle, WA USA; 10 Epidemiology Program, Division of Public Health Sciences, Fred Hutchinson Cancer Center, Seattle, WA USA

**Keywords:** Geospatial analyses, Vulnerable populations, Environmental justice

## Abstract

**Background:**

Aircraft noise is a key concern for communities surrounding airports, with increasing evidence for health effects and inequitable distributions of exposure. However, there have been limited national-scale assessments of aircraft noise exposure over time and across noise metrics, limiting evaluation of population exposure patterns.

**Objective:**

We evaluated national-scale temporal trends in aviation noise exposure by airport characteristics and across racial/ethnic populations in the U.S.

**Methods:**

Noise contours were modeled for 90 U.S. airports in 5-year intervals between 1995 and 2015 using the Federal Aviation Administration’s Aviation Environmental Design Tool. We utilized linear fixed effects models to estimate changes in noise exposure areas for day-night average sound levels (DNL) of 45, 65, and a nighttime equivalent sound level (L_night_) of 45 A-weighted decibels (dB[A]). We used group-based trajectory modeling to identify distinct groups of airports sharing underlying characteristics. We overlaid noise contours and Census tract data from the U.S. Census Bureau and American Community Surveys for 2000 to 2015 to estimate exposure changes overall and by race/ethnicity.

**Results:**

National-scale analyses showed non-monotonic trends in mean exposed areas that peaked in 2000, followed by a 37% decrease from 2005 to 2010 and a subsequent increase in 2015. We identified four distinct trajectory groups of airports sharing latent characteristics related to size and activity patterns. Those populations identifying as minority (e.g., Hispanic/Latino, Black/African American, Asian) experienced higher proportions of exposure relative to their subgroup populations compared to non-Hispanic or White populations across all years, indicating ethnic and racial disparities in airport noise exposure that persist over time.

**Significance:**

Overall, these data identified differential exposure trends across airports and subpopulations, helping to identify vulnerable communities for aviation noise in the U.S.

**Impact statement:**

We conducted a descriptive analysis of temporal trends in aviation noise exposure in the U.S. at a national level. Using data from 90 U.S. airports over a span of two decades, we characterized the noise exposure trends overall and by airport characteristics, while estimating the numbers of exposed by population demographics to help identify the impact on vulnerable communities who may bear the burden of aircraft noise exposure.

## Introduction

There has been long-term growth in aircraft passenger boardings (enplanements) in the U.S., with a 40% increase from over 526 to 738 million passengers between 1995 and 2015 [1]. To meet the demand and improve aviation performance, there has been continual interest in the expansion and development of airports. However, the noise generated by aircraft landing and take-off (LTO) operations is a key challenge facing many communities surrounding airports. Despite advancements in technologies and federal noise standards that have resulted in fewer, more efficient LTO operations and quieter aircraft [2], noise complaints and annoyance have been shown to be associated with aircraft noise exposure [3], and continue to increase for airport-adjacent communities [4].

Aircraft noise levels have been shown to have adverse health effects, leading to efforts at regulation globally [5]. Noise-related health effects include annoyance [6, 7], impaired learning in children [8, 9], speech interference, sleep disturbance [10, 11], adverse birth outcomes [12, 13] and increased cardiovascular risk factors (e.g., hypertension) [14, 15] and disease [16–18].

The most recent analysis of U.S. population exposure to aircraft noise was from 2000–2010 at levels as low as day-night average sound level (DNL) 55 A-weighted decibels (dB[A]) [19]. Another national-scale estimation of numbers of people with significant noise exposure (DNL 65 dB[A]) showed a general decrease over time from seven million in 1972 down to 292,000 in 2010 [20]. While these nationwide assessments capture the impact of noise exposure at a broader population level, they have been limited in exploring whether there are inequitable distributions across sub-populations and do not capture lower noise exposures that may be relevant to community concerns and the health effects of noise. Some studies have shown higher burdens of aircraft noise exposure among vulnerable or marginalized groups, albeit analyzed around a single U.S. airport or for limited years [21–25].

We therefore assessed national spatiotemporal trends in aircraft noise exposure in the U.S. from 1995–2015. We provide findings by airport characteristics, sociodemographic characteristics of exposed populations, and combined airport and sociodemographic characteristics. We also expand upon the time span, decibel range, and relevant noise metrics used in previous assessments. Our evaluation of the magnitude, breadth, and impact of aircraft noise exposure within the U.S. and identification of specific populations at high risk of exposure may inform analyses assessing noise and health that may be critical to guiding stakeholders, such as legislators, industry partners, and community groups invested in the development of aircraft noise policy.

## Methods

To investigate aircraft noise exposure trends by airport and populations exposed, we utilized noise exposure contours for U.S. airports, identified airport characteristics, and estimated exposed populations by race and ethnicity.

### Noise assessment

**Fig. 1:**
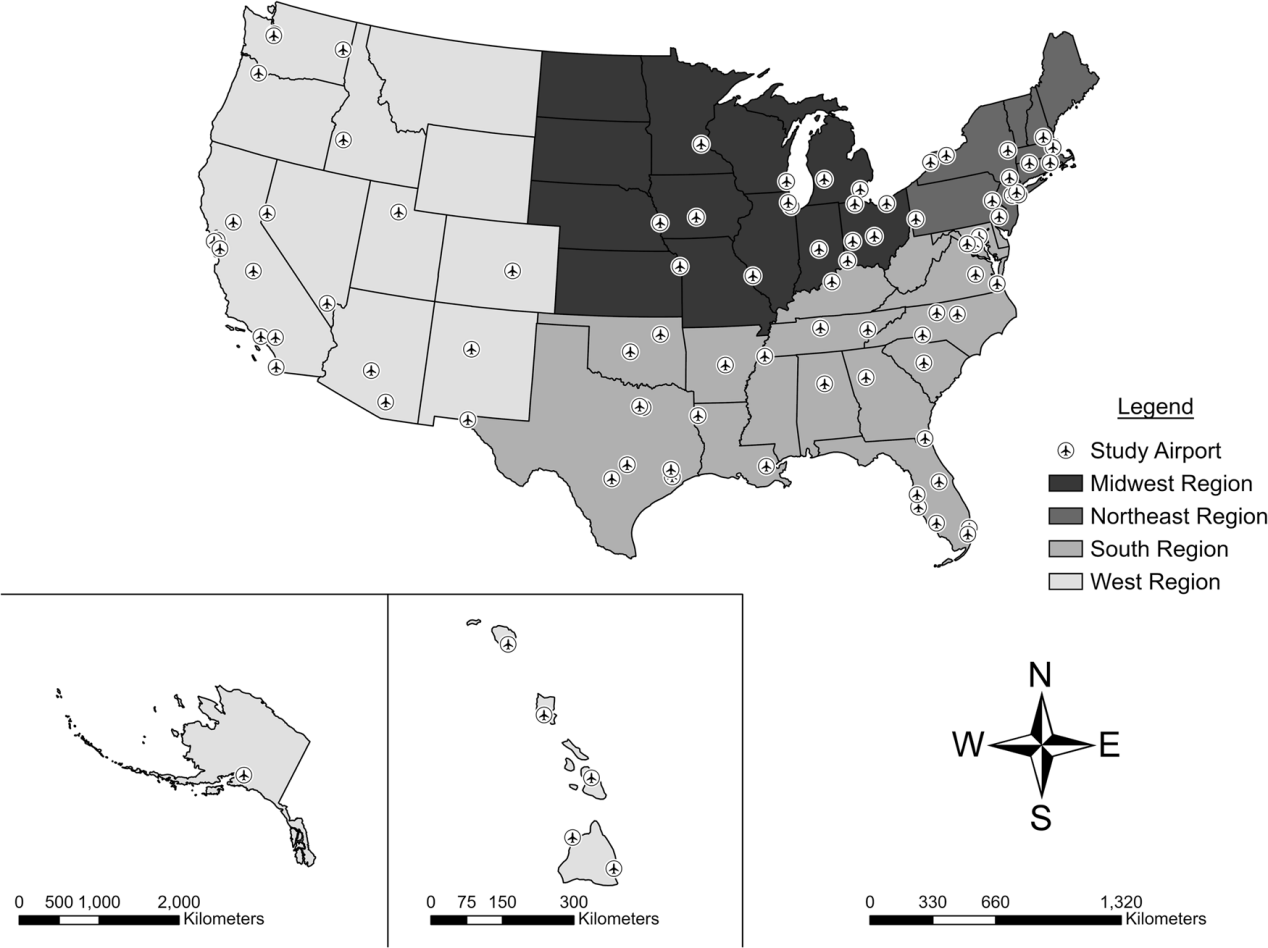
Sample of U.S. airports (*n* = 90) included in the study.

We obtained noise exposure contours for 90 U.S. airports (Fig. 1) from the U.S. Department of Transportation (DOT) Volpe National Transportation Systems Center (Volpe). The airports included in this study constitute 18% of the Part 139 U.S. Federal Aviation Administration (FAA) certified airports, but represent 88% of total enplanements in 2015 [26]. Noise contours were modeled for years 1995, 2000, 2005, 2010, and 2015. Detailed information on the generation of aircraft noise contours is provided elsewhere [24, 27]. Briefly, noise contours were created using FAA’s Aviation Environmental Design Tool (AEDT), which was developed using internationally accepted practices to estimate the environmental impact of aviation. Estimations were formulated with data (e.g., airport runway locations and utilization) from the Enhanced Traffic Management System (ETMS) for 2000–2015 and Official Aviation Guide (OAG) for 1995 and standard aircraft profile data in the Aircraft Noise and Performance (ANP) database.

We used two noise metrics in this study: DNL and L_night_. DNL reflects noise exposure for an average 24-h period of the year that artificially penalizes nighttime hours by adding 10 dB(A) to measurements from 22:00–07:00 when noise sensitivity may be higher due to lower ambient noise. L_night_ reflects noise exposure summarized over nighttime hours. DNL and L_night_ were modeled in one dB(A) increments ranging from 45–75 dB(A).

We focused on three noise thresholds: (1) DNL 65, (2) DNL 45, and (3) L_night_ 45 dB(A) levels, the first of which relates to the U.S. regulatory threshold for significant noise exposure, and the latter two of which correspond to the World Health Organization recommended guidelines for aircraft noise exposure in the European region [5]. Our nighttime threshold is limited to the lowest available modeled data at 45 dB(A).

To exclude non-livable areas from the assessments, we overlaid the contours with national area hydrography (i.e., water bodies) and greenspace geodatabases. National area hydrography geodatabases (ponds, lakes, oceans, swamps, glaciers, rivers, streams, and/or canals) were available from the U.S. Census Topologically Integrated Geographic Encoding and Referencing (TIGER) database. Hydrography databases for 2013 [28] were overlaid with 1995–2010 noise contours, and for 2016 [29] with 2015 contours. A 2010 national greenspace layer (parks, gardens, and forests) was available from Esri [30] and overlaid with all contour years.

### Airport characteristics

We identified various airport characteristics by the four U.S. Census regions (Midwest, Northeast, South and West) and FAA hub type designation from 2001 (passenger/cargo airline hub type, and cargo hub). FAA categorizes primary commercial airports (more than 10,000 passenger boardings each year) into hubs according to 49 U.S. Code § 47102, where large hubs receive greater than or equal to 1% of the annual U.S. commercial enplanements, medium hubs 0.25–1%, small hubs 0.05–0.25%, and nonhubs less than 0.05% but more than 10,000 passenger boardings per year [31]. Passenger/cargo airline hub type was categorized according to mainline passenger and cargo airline designations of airports. Concentrated LTO operations use hub-and-spoke, where airlines centralize regional operations to major central hubs, or point-to-point models, which are direct A–B operations that do not require passing through a central hub [32]. Airports were designated as primary if serving as a main central hub for a hub-and-spoke airline, secondary if serving as a support hub for a hub-and-spoke airline, focus city if designated as a focal airport for a point-to-point airline, or nonhub/focus city if not serving as a hub or focus city. We categorized airports as a cargo hub if ranked by the FAA as among the top 25 for all-cargo landed weights. Airport passenger enplanement and cargo data were available from the Bureau of Transportation Statistics for 1995 and from the FAA Air Carrier Activity Information System (ACAIS) database for 2000–2015 [33, 34]. LTO operations data were available from the Air Traffic Activity System (ATADS) database for 1995–2015 [35].

### Analysis of trends in airport characteristics

We first estimated mean changes in contour areas over time using response profile analyses across all 90 airports. The analysis of response profiles allows for characterizations of patterns of change in the mean contour area over time. This method is appropriate for longitudinal studies with a balanced design, when timing of repeated measures are uniform across subjects, and for data that violate assumptions of independence and homogeneity of variance [36]. Contour area data were complete for all 90 airports across each study time-point and were assumed to correlate across years by airport. Covariance structures were selected by examining fit statistics tables and likelihood ratio tests for nested models.

Rather than solely relying on fixed, a priori factors, identifying distinct groups of airports with shared characteristics could provide an informed approach for epidemiological studies to utilize different airport characteristics in exploring associations between aircraft noise exposure and health. We assessed variation between airports by statistically arranging airports by similarity using group-based trajectory modeling (GBTM). GBTM is a specialized application of finite mixture models that identifies distinct groups sharing underlying characteristics and trajectories [37]. We applied the SAS package *Proc Traj* with a beta regression, which is appropriate for non-normal distributions [38, 39]. The beta distribution dictates normalizing noise contour areas to fit within a zero to one range using the minimum and maximum area values within respective years [40]. Model parameters were estimated using maximum likelihood. To determine the optimal number of groups, we started with a one-group model and sequentially fitted an increasing number of groups in a stepwise manner. The best fitting model was selected using the following criteria: logged Bayes factor (2Δ BIC), Jeffreys’ scale of evidence for Bayes factors, non-overlapping confidence intervals, a posterior-probability of group membership greater than 0.7, and approaching a sufficient sample size of ideally ≥5% in each group [41, 42]. We simultaneously determined the shape of each trajectory over time (i.e., order of a polynomial relationship) using BIC values. We tested for nonrandom associations between characteristics and trajectory groups using Fisher’s exact test due to small cell sizes.

### Analysis of trends in exposed population

We evaluated changes in exposed populations overall, by Hispanic/Latino ethnicity, and race as defined by the U.S. Census. Using the U.S. Census designation, Hispanic/Latino ethnicity was categorized as those who identify as Hispanic or Latino versus non-Hispanic/non-Latino. Race was categorized as those who identified as White alone, Black or African American alone, Asian alone, American Indian/Alaska Native, Native Hawaiian/Other Pacific Islander alone, some other race alone, or two or more races. Population data were obtained at the Census tract level for 2000, 2005, 2010, and 2015 from GeoLytics Inc. After 2001, inter-decennial Census categorizations for race excluded “some other race” and reapportioned “some other race” and part of “two or more races” into remaining races. For consistency of race categories over time we used race counts from the decennial Census for 2000 and 2010 and the 5-year American Community Survey (ACS) estimates for 2005 and 2015. All Census and ACS data were aligned at the 2010 census tract boundaries for comparative analysis from 2000–2015. Data for year 2000 and 2015 were available from GeoLytics preweighted to 2010 boundaries, while 2005 data were interpolated to 2010 boundaries using geographic crosswalks available from IPUMS National Historical Geographic Information System [43]. We estimated number of people residing in areas of noise exposure using simple area weighting, which sums the proportions of masked noise contour areas that overlap with tracts multiplied by the population estimates within overlapping tracts.

Exposed population estimates were evaluated in the following ways: (1) normalized by the tracts’ respective sub-population; (2) by absolute counts; and (3) normalized by the tracts’ total population. Tracts were selected (*n* = 13,416) if they intersected the largest noise exposure contour during our study period (DNL 45, dB[A] for 2000); we defined these tracts as “living close to airports”. We normalized by tract sub-populations to assess whether there was a disproportionate burden of exposure on racial/ethnic groups (e.g., exposed Hispanic/Latino population normalized by the total Hispanic/Latino population living within the tracts around the airport), and normalized by the tract total population in order to account for overall changes in population growth/decline.

### Analysis of trends in exposed populations by airport characteristics

In order to determine if the sociodemographic characteristics of the population exposed to aircraft noise differed by airport characteristics over time, we also examined changes in counts and normalized proportions of exposed populations when stratified by trajectory groups. We hypothesized that this analysis would provide insight into the association between the shared underlying properties determining aircraft noise exposure trajectories and demographic characteristics, such as ethnicity and race, of exposed populations.

Spatial analyses were completed using a common projected coordinate system within a geographic information system (GIS; Esri ArcGIS® Pro V2.2.3; Redlands, California). Geographic areas were estimated in units of square kilometers (km^2^) after masking. Statistical analyses were performed using Statistical Analysis System (SAS) v9.4 (Cary, North Carolina).

## Results

Table 1 presents the distribution of airport characteristics for the 90 U.S. airports with modeled noise contours. The largest numbers of airports were located in the South region (38.9%), were medium FAA hubs (35.6%), were in nonhub/focus cities (47.8%), and were non-cargo hubs (73.3%).

**Table 1:** Characteristics of study sample of U.S airports.

Characteristic	*n*	%
Airports	90	100.0
Region		
Midwest	15	16.7
Northeast	14	15.6
South	35	38.9
West	26	28.9
Federal Aviation Administration Hub Type		
Large	30	33.3
Medium	32	35.6
Small	24	26.7
Nonhub	4	4.4
Passenger/Cargo Airline Hub		
Primary	5	5.6
Secondary	25	27.8
Focus City	17	18.9
Nonhub/Focus City	43	47.8
Cargo Hub		
Yes	24	26.7
No	66	73.3

### Trends in noise contour areas

**Fig. 2:**
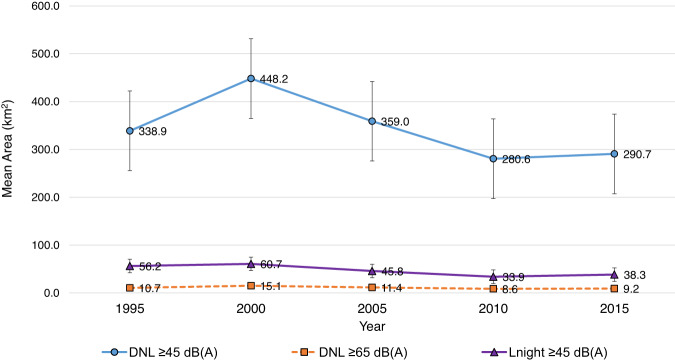
Temporal trends in mean aircraft noise contour size for 90 U.S. airports. dB(A) A-weighted decibels, DNL day–night average sound level, L_night_ nighttime equivalent sound level.

In determining trends in areas exposed to noise, unstructured covariance patterns showed the best fit in our evaluation of the mean response profiles of the 90 airports. Average areas found within DNL 45 dB(A) noise contours for the 90 U.S. airports peaked in 2000, followed by a 37% decrease to 2010 and 4% increase from 2010 to 2015 (Fig. 2). For the 90 U.S. airports included in this study, annual passenger enplanements increased from 487 to 702 million from 1995–2015, whereas LTO operations decreased from 246,000 to 192,000. Trends were similar for DNL 65 and L_night_ 45 dB(A) (Supplemental Fig. 1).

We statistically grouped study airports by their shared underlying characteristics for DNL 45, 65 and L_night_ 45 dB(A) thresholds using GBTM. Our data revealed the best fit with four distinct trajectory groups and cubic functions. Based on these trajectory group rankings of noise contour areas, we labeled the highest noise trajectory groups as *extra-large*, second highest as *large*, third highest as *medium*, and lowest as *small* (Supplemental Fig. 2a, b, c). Extra-large trajectory groups were mostly comprised of large FAA hubs, primary passenger/cargo airline hubs, and cargo hubs (Supplemental Table 1). Large trajectory groups were typically large hubs but were mostly comprised of secondary passenger/cargo airline hubs. We found divergent characteristics for DNL 65 dB(A) contours when assessing the medium trajectory groups. Medium trajectory groups for DNL 45 and L_night_ 45 dB(A) tended to be medium FAA hubs, nonhub/focus city passenger/cargo airline hubs, and non-cargo hubs, whereas for DNL 65 dB(A), the medium trajectory group tended to be large FAA hubs, secondary passenger/cargo airline hubs, and cargo hubs (similar to the large trajectory group though with a notable geographic difference). Small trajectory groups for all metric/dB(A) levels tended to be nonhub/focus cities and non-cargo hubs; however, DNL 45 and L_night_ 45 dB(A) tended to be small FAA hubs whereas DNL 65 dB(A) was mostly comprised of medium/small FAA hubs. We found that trajectory groups were significantly associated with categories of FAA hub, passenger/cargo airline hub, and cargo hub for each metric/dB(A) level (*p* < 0.05), indicating that these airport characteristics were not independent of trajectory group assignment.

In assessing trajectory group trends (Supplemental Fig. 2), extra-large, large, and medium trajectory groups had patterns consistent with overall trends of noise contour areas for all 90 airports for DNL 45, 65 and L_night_ 45 dB(A). On the other hand, small trajectory groups for DNL 65 dB(A) had a slight increasing trend after 2010 but decreased after 2010 for DNL and L_night_ 45 dB(A).

### Trends in exposed populations

**Fig. 3:**
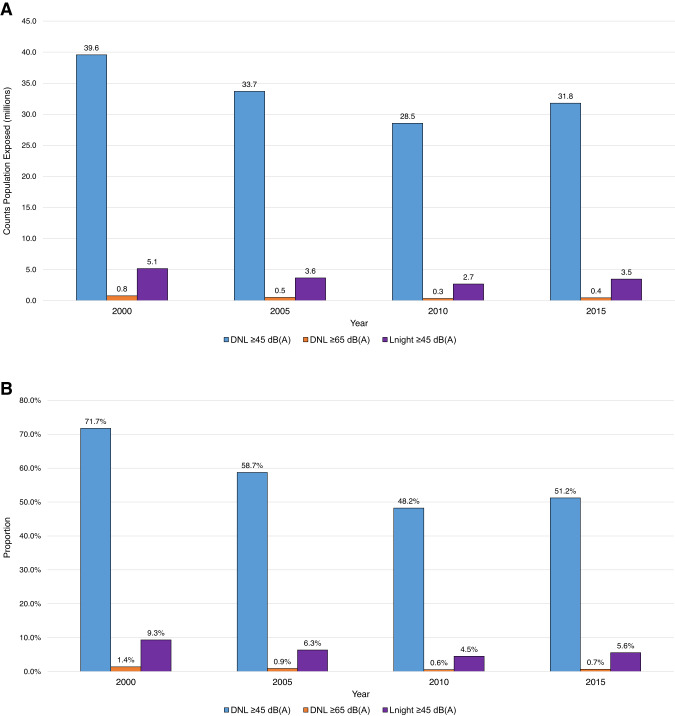
Temporal trends in residents exposed to aircraft noise around 90 U.S. airports. **A** Total counts and **B** normalized by tract total population^a^. ^a^Normalized by tract total population: denominator is the total population (e.g., no. of total population exposed in tracts / no. of total population living in tracts around airports). dB(A) A-weighted decibels, DNL, day–night average sound level, L_night_ nighttime equivalent sound level.

The total population living within noise exposure contours around our 90 U.S. airports peaked in 2000, decreased in 2005 and 2010, and increased from 2010 to 2015 across all noise metric/dB(A) levels (Fig. 3A). Normalizing by total tract population showed consistent trends with those seen for counts (Fig. 3B).

**Fig. 4:**
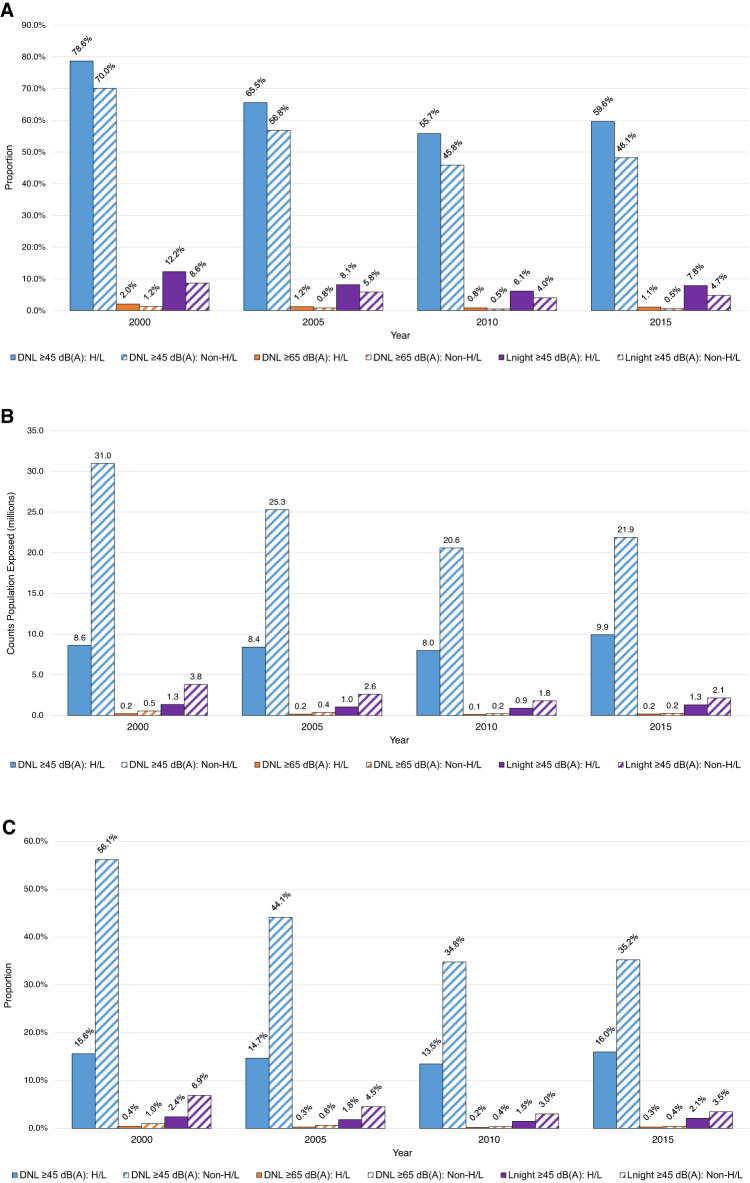
Temporal trends in residents exposed to aircraft noise around 90 U.S. airports by Hispanic/Latino status. Presented as **A** normalized by tract Hispanic/Latino or non-Hispanic/Latino populations^a^, **B** total counts, and **C** normalized by tract total population^b^. ^a^Normalized by tract sub-population: denominator is the subgroup population (e.g., no. of Hispanics exposed in tracts / no. of Hispanics living in tracts around airports). ^b^Normalized by tract total population: denominator is the total population (e.g., no. of Hispanics exposed in tracts / no. of total population living in tracts around airports). dB(A) A-weighted decibels, DNL day–night average sound level, H/L Hispanic/Latino, L_night_ nighttime equivalent sound level.

**Fig. 5:**
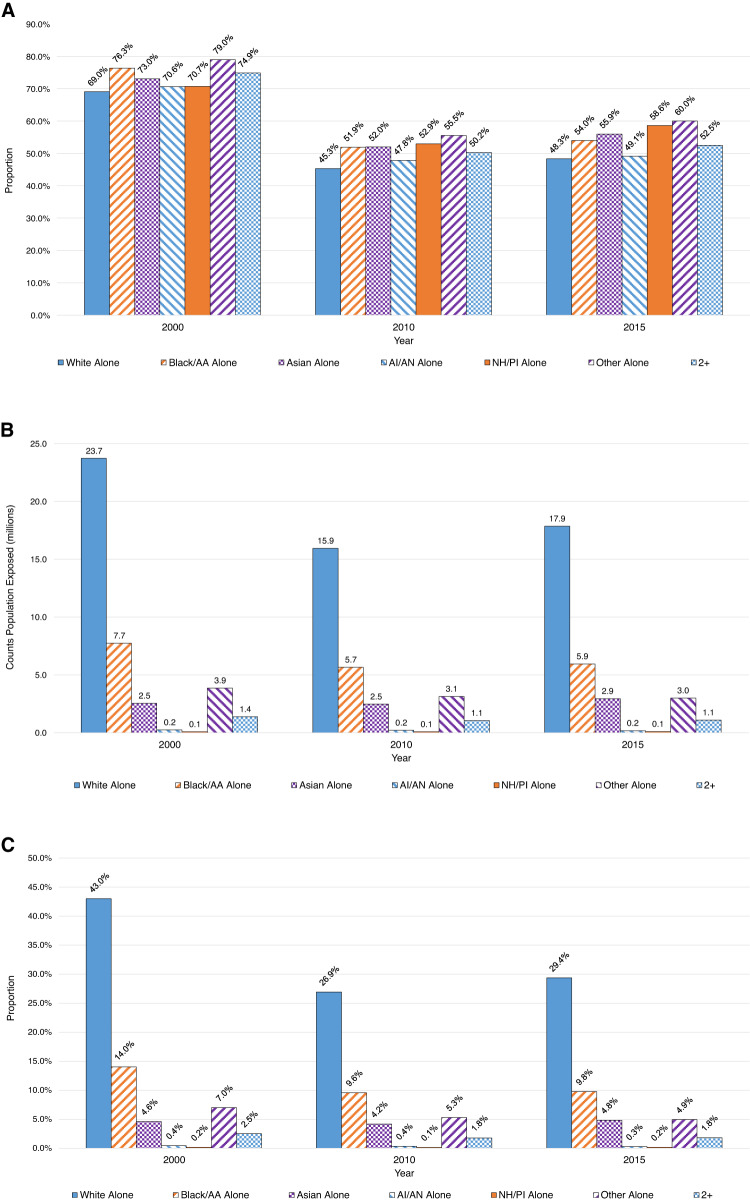
Temporal trends in residents exposed to aircraft noise around 90 U.S. airports by race. Presented as **A** normalized by tract race group population^a^, **B** total counts, and **C** normalized by tract total population^b^. Note: Lacking 2005 race data due to inconsistent reapportionment of “Other Alone”. ^a^Normalized by tract sub-population: denominator is the subgroup population (e.g., no. of White alone exposed in tracts / no. of White alone living in tracts around airports). ^b^Normalized by tract total population: denominator is the total population (e.g., no. of White alone exposed in tracts / no. of total population living in tracts around airports). AA African American, AI/AN American Indian/Alaska Native, dB(A) A-weighted decibels, DNL day–night average sound level, L_night_ nighttime equivalent sound level, NH/PI Native Hawaiian/Pacific Islander, 2+ two or more races.

After normalizing by tract Hispanic/Latino sub-populations, we found that greater proportions of Hispanic/Latino residents lived within DNL 45 dB(A) noise exposure contours compared to non-Hispanic/Latino residents (Fig. 4A); this finding was consistent across years and metric/dB(A) levels. For example, while 79% of Hispanics/Latinos in the study areas lived within the maximum spatial extent of the DNL 45 dB(A) contours in 2000, only 70% of non-Hispanics/Latinos did. Similarly, after normalizing by tract respective race sub-populations, we observed that each non-White race group living within DNL 45 dB(A) areas had greater proportions of exposure compared to those who identified as White (Fig. 5A). Overall counts and proportions normalized by total tract population for Hispanic/Latino ethnicity and race groups are presented in Figs. 4B, 4C and Figs. 5B, 5C, respectively.

Trends for overall counts, normalized by total tract population and by tract sub-populations, were consistent for DNL 65 and L_night_ 45 dB(A) (Supplemental Figs. 3 and 4, respectively). When stratifying by trajectory groups, we found that the disproportionate burden on Hispanic and non-White populations persisted across all four trajectory groups and over all study years (Supplemental Tables 2a-c).

## Discussion

This study leveraged longitudinal noise contour data for 90 U.S. airports to expand our understanding of how aircraft noise exposure and the populations exposed have changed over time. Our data revealed non-monotonic trends in noise contour areas over time, reflecting the combination of changes in aircraft operations, technology, and airport utilization. LTO operations were influenced by events such as the terrorist attacks of 9/11 in 2001, the 2003 Severe Acute Respiratory Syndrome (SARS) pandemic, and the Great Recession of 2008, all of which heavily impacted the aviation industry [44, 45]. The general trend of a steep climb in passenger enplanements and decrease in LTO operations likely reflects the rising efforts towards economic growth and efficiency for air travel in the time before the COVID-19 pandemic.

Further, we identified similar (but not identical) trends over time across four distinct groups among our study airports that shared underlying characteristics determining aircraft noise exposure areas. The extra-large trajectory group consisted of the main hubs for the largest mainline passenger and freight airlines in the U.S. These airports act as the major central hub in the hub-and-spoke model and reflect the concentration of airline fleets for transit and maintenance [32]. Cargo hubs were predominantly seen in the largest trajectory groups for each metric and decibel level. Cargo operations mostly occur during nighttime hours in order to prioritize commercial flights during daytime hours. While L_night_ may indicate the influence of nighttime cargo operations, DNL is an averaged metric for a 24-h period wherein, even with the artificial 10 dB(A) penalty for nighttime hours, the effect of nighttime operations may attenuate when averaged with daytime operations. In all, passenger/cargo airline hub may be a predominant, although incomplete, airport characteristic that explains trajectory group membership. This factor likely reflects operations and fleet mix, which are influential factors on the extent of noise contours. Overall, our ability to group airports based on underlying characteristics may prove useful in epidemiological investigations of noise and health, e.g. in utilizing propensity score matching within trajectory groups to obtain comparability between exposed and unexposed participants with respect to observed characteristics, identifying population subgroups where the effects of an intervention may vary, or framing possible biases from unmeasured confounders relating to groups [46].

In addition, we found that greater proportions of Hispanic/Latino, Black/African American, and Asian populations were more likely to live in areas of high exposure compared to non-Hispanic/Latino or White residents. These trends persisted across time and over airport trajectory groups. In a recent study exploring sociodemographic characteristics of populations exposed to aircraft noise using the same exposure data in this present study for 2010, block groups with a higher Hispanic population had greater odds of being exposed to aircraft noise [24]. Also, Casey et al. [21] found evidence of disproportionate environmental noise exposure throughout the U.S. in 2010 using 2006–2010 ACS block group data, with greater estimated day and nighttime noise levels for block groups with higher proportions of non-White residents. Other studies have shown that airport-adjacent communities have elevated percentages of minority and low-income populations [23, 47, 48]. As such, our study results mirror that of previous literature, adding the longitudinal perspective to reinforce the patterns of disparities over time.

Our findings emphasize the importance of accounting for the unique underlying characteristics of airports that influence how noise exposure changes over time, with consideration of multiple noise metrics. Although not explored in this study, these trends have been altered by the COVID-19 pandemic with a period of global travel bans and diminished demand. We hypothesize that travel bans and general reductions in travel demand during the pandemic, as an external driver, would generally shrink aircraft noise contours and subsequently decrease populations exposed. However, while DNL exposures would likely have been reduced, L_night_ may have witnessed an initial decline followed by an increase due to greater demand for the transit goods and supplies necessary for the response and lifestyle adaptation to the pandemic. Demand in air cargo transportation has remained stable throughout the pandemic and air travel is expected to recover over the course of two to four years post-pandemic [49]. Future work should therefore isolate and validate the effects of the pandemic across noise metrics and airport types, with an explicit evaluation of the effect of transportation of cargo on nighttime community noise exposure.

Our study presented a few limitations. First, we used Census tract data to estimate the population living within noise exposure contours, which may be a source of bias due to the modifiable areal unit problem (MAUP) and selective aggregation of population counts within a given boundary. One method of addressing this limitation is to perform area weighting using population estimates at a finer spatial resolution (i.e., block groups). However, the Census tract level was selected since block group estimates are prone to greater measurement error and for continuity of data across all years. Second, our study incorporated data from a non-random sample of 90 U.S. airports. Airports were selected based on availability of operations data for study years [34]. Nevertheless, this sample of major airports was able to capture the majority of passenger enplanements and encompass the variety of characteristics that influence noise exposure over time. Third, the ideal number of trajectory groups found using GBTM was not immutable; in other words, it was reflective of the availability of our data in that analyzing an alternative sample of airports or noise exposure time-points (e.g., annual versus 5-year) would likely alter the number of groups. Also, interpretation of the underlying airport characteristics shared by each trajectory group was observational and could vary by decibel level and metrics used, which may complicate applications to population research. Still, this statistical tool provides insight and is informative of the latent characteristics shared by our sample of airports. Fourth, we focused on DNL as a principal metric to describe exposure, but some have argued that it may not accurately capture the experience of noise exposure and is not easily understood by the general public and stakeholders who rely on exposure metrics for noise abatement and mitigation efforts [50, 51]. In 2010, the FAA implemented the Next Generation Air Transportation System (NextGen), a nationwide modernization effort for the U.S. airspace infrastructure that incorporates navigational technologies offering precise and efficient procedures to reduce flying time, fuel usage, and aircraft exhaust emissions [52]. In doing so, aircraft departure and arrival patterns have transitioned to narrow routes that may yield shrinking noise contours and fewer residents exposed; however, those living underneath the new “highways in the sky” would be inundated with more frequent exposure events and potential increases in exposure, making the net implications of various noise metrics unclear. Therefore, future studies may consider studying the effects of NextGen, particularly on communities carrying the burden of exposure, as well as incorporating alternative metrics that may provide a more comprehensive picture of aircraft noise exposure when assessed in conjunction with DNL. For example, exposure may incorporate peak-DNL or sound exposure levels, or number of flight events.

In spite of these limitations, our study offers valuable insight about aircraft noise exposure patterns over time in the U.S. Strengths of our study include the availability of noise exposure contours for 90 U.S. airports across 20 years, using the same underlying model and population assumptions across airports and time. In addition, we were able to examine patterns for metrics beyond DNL, including an assessment of trends for nighttime noise exposure which may be more relevant for sleep health outcomes or outcomes connected with sleep disturbance (e.g., hypertension).

## Conclusion

In conclusion, we found that aircraft noise exposure from 1995–2015 for 90 U.S. airports generally peaked in 2000, decreased to 2010, and increased to 2015. Our sample of airports could be categorized into distinct groups sharing underlying characteristics (e.g., FAA or passenger/cargo airline hub designation or being a major cargo airport) that may be determinative of noise exposure trends over time. Minority populations (e.g., Hispanic/Latino, Black/African American, Asian) were proportionally more likely to live in areas of elevated exposure over time and across airports, highlighting the disproportionate burden of this environmental hazard.

## Data Availability

Noise contour data used in supporting the findings of this study were used under a data use agreement with the U.S. Federal Aviation Administration and are available for use for research purposes as outlined in the agreement. All other data analyzed in this study are publicly available from referenced sources, or available for purchase from Geolytics Inc.

## Supplementry Informaton

Supplementry Informaton
