# Supplementary material for: Characterizing temporal trends in populations exposed to aircraft noise around U.S. airports: 1995–2015
Source: J Expo Sci Environ Epidemiol. 2023 Sep 21;35(1):70–9. doi: 10.1038/s41370-023-00575-5 (PMC11876072; doi:10.1038/s41370-023-00575-5)
Supplement: Supplementary file 1 — Supplementry Informaton [file 41370_2023_575_MOESM1_ESM.pdf]

Supplementary Information

Characterizing temporal trends in populations exposed to aircraft noise around U.S. airports: 1995-2015

Supplemental Table 1. Characteristics of study airports (n=90) by trajectory group membership.[a]

Characteristic	DNL ≥45 dB(A)				DNL ≥65 dB(A)				L_{night} ≥45 dB(A)			
	S	M	L	XL	S	M	L	XL	S	M	L	XL
Airports, %	28.9	40.0	26.7	4.4	65.6	24.4	5.6	4.4	33.3	35.6	25.6	5.6
Region, %												
Midwest	11.5	13.9	25.0	25.0	13.6	27.3	0.0	25.0	10.0	15.6	26.1	20.0
Northeast	26.9	8.3	16.7	0.0	17.0	9.1	40.0	0.0	26.7	9.4	13.0	0.0
South	30.8	44.4	33.3	75.0	39.0	36.4	20.0	75.0	30.0	46.9	34.8	60.0
West	30.8	33.3	25.0	0.0	30.5	27.3	40.0	0.0	33.3	28.1	26.1	20.0
FAA Hub Type, %[b]												
Large	0.0	25.0	75.0	75.0	10.2	72.7	100.0	75.0	3.3	21.9	78.3	80.0
Medium	19.2	55.6	25.0	25.0	42.4	27.3	0.0	25.0	20.0	62.5	21.7	20.0
Small	65.4	19.4	0.0	0.0	40.7	0.0	0.0	0.0	63.3	15.6	0.0	0.0
Nonhub	15.4	0.0	0.0	0.0	6.8	0.0	0.0	0.0	13.3	0.0	0.0	0.0
Passenger/Cargo Airline Hub, %[b]												
Primary	0.0	0.0	4.2	100.0	0.0	4.6	0.0	100.0	0.0	0.0	4.4	80.0
Secondary	11.5	19.4	62.5	0.0	11.9	63.6	80.0	0.0	16.7	12.5	65.2	20.0
Focus City	15.4	19.4	25.0	0.0	18.6	22.7	20.0	0.0	13.3	28.1	17.4	0.0
Nonhub/Focus City	73.1	61.1	8.3	0.0	69.5	9.1	0.0	0.0	70.0	59.4	13.0	0.0
Cargo Hub, %[b]												
Yes	7.7	13.9	54.2	100.0	6.8	54.6	80.0	100.0	6.7	12.5	56.5	100.0
No	92.3	86.1	45.8	0.0	93.2	45.5	20.0	0.0	93.3	87.5	43.5	0.0

Note: S = lowest noise contour area trajectory group; M = third highest noise contour area trajectory group; L = second noise contour area trajectory group; XL = highest noise contour area trajectory group.

[a] Percentages by column

[b] p-value <0.05 for association between airport characteristics and trajectory groups.

Abbreviations: DNL, day-night average sound level; FAA, Federal Aviation Administration; L, large; L_{night}, nighttime equivalent sound level; M, medium; S, small; XL, extra-large.

Supplemental Table 2. Counts and normalized proportions for total, Hispanic/Latino ethnicity, and race group populations stratified by trajectory group and residing in areas exposed to a) DNL 45, b) DNL 65, and c) L_{night} 45 dB(A).

a)

	Year	Normalized by Tract H/L or non-H/L Population[a] (%)				Counts (millions)				Normalized by Tract Total Population[b] (%)			
		2000	2005	2010	2015	2000	2005	2010	2015	2000	2005	2010	2015
Population	Group												
Total	S					2.89	2.60	1.87	1.77	53.2	46.2	31.5	28.6
	M					15.00	11.98	10.36	11.94	71.8	55.1	45.9	49.8
	L					15.97	13.96	12.10	13.74	73.4	61.5	51.7	56.1
	XL					5.71	5.13	4.22	4.34	80.7	70.8	58.0	58.5
H/L	S	57.3	51.1	34.1	35.4	0.28	0.30	0.26	0.31	5.2	5.4	4.4	5.0
	M	79.5	63.2	53.4	56.0	4.42	3.98	3.69	4.46	21.1	18.3	16.3	18.6
	L	78.1	68.4	60.2	66.6	2.81	3.02	3.00	3.91	12.9	13.3	12.8	16.0
	XL	84.2	72.3	62.0	64.2	1.08	1.10	1.01	1.23	15.2	15.1	13.9	16.6
Non-H/L	S	52.8	45.6	31.1	27.4	2.61	2.30	1.61	1.46	48.0	40.8	27.1	23.5
	M	69.0	51.8	42.5	46.7	10.58	8.00	6.67	7.48	50.7	36.8	29.5	31.2
	L	72.4	59.8	49.4	52.8	13.15	10.94	9.09	9.83	60.4	48.2	38.9	40.2
	XL	79.9	70.4	56.8	56.6	4.63	4.04	3.21	3.11	65.4	55.7	44.0	42.0
White Alone	S	51.1		29.9	27.0	1.92		1.15	1.07	35.3		19.4	17.6
	M	68.9		43.3	47.1	8.56		5.69	6.57	41.0		25.2	28.0
	L	70.9		48.2	52.7	9.81		6.76	7.77	45.1		28.9	32.4
	XL	79.0		56.1	56.6	3.43		2.35	2.45	48.5		32.2	33.5
Black/AA Alone	S	60.3		35.7	28.3	0.46		0.31	0.25	8.5		5.2	4.1
	M	74.1		45.2	49.0	2.50		1.60	1.74	12.0		7.1	7.4
	L	78.5		57.1	60.3	3.49		2.74	2.93	16.0		11.7	12.2

	XL	82.9	59.7	59.7	1.28	1.02	1.02	18.2	14.0	13.9
Asian Alone	S	55.5	33.3	34.9	0.25	0.20	0.23	4.7	3.4	3.7
	M	75.5	53.8	59.0	1.30	1.22	1.48	6.2	5.4	6.3
	L	73.1	54.2	58.8	0.68	0.76	0.92	3.1	3.2	3.9
	XL	83.5	60.5	59.2	0.30	0.29	0.31	4.3	4.0	4.3
AI/AN Alone	S	55.8	34.5	33.1	0.01	0.01	0.01	0.3	0.2	0.2
	M	69.2	44.1	46.3	0.11	0.09	0.07	0.5	0.4	0.3
	L	73.3	52.3	53.9	0.10	0.09	0.08	0.5	0.4	0.3
	XL	81.4	60.3	60.3	0.02	0.02	0.01	0.3	0.3	0.2
NH/PI Alone	S	48.3	32.9	30.6	0.01	0.01	0.01	0.2	0.2	0.1
	M	73.9	54.7	65.0	0.05	0.04	0.05	0.2	0.2	0.2
	L	77.1	60.7	65.3	0.02	0.03	0.03	0.1	0.1	0.1
	XL	86.1	63.1	58.6	0.00	0.00	0.00	0.1	0.0	0.0
Other Alone	S	58.9	34.0	36.5	0.14	0.11	0.10	2.6	1.9	1.7
	M	79.8	52.0	54.7	1.82	1.28	1.28	8.7	5.7	5.4
	L	78.7	60.5	68.3	1.38	1.31	1.25	6.3	5.6	5.2
	XL	84.3	62.7	66.6	0.52	0.44	0.37	7.3	6.0	5.0
2+	S	53.5	33.6	31.4	0.09	0.08	0.08	1.7	1.3	1.3
	M	76.6	48.8	53.1	0.66	0.44	0.47	3.2	2.0	2.0
	L	75.9	54.4	57.1	0.49	0.42	0.44	2.2	1.8	1.9
	XL	83.7	60.0	58.6	0.14	0.11	0.10	2.0	1.5	1.3

b)

Population	Group	Normalized by Tract H/L or non-H/L Population[a] (%) 2000	2005	2010	2015	Counts (millions) 2000	2005	2010	2015	Normalized by Tract Total Population[b] (%) 2000	2005	2010	2015
Total	S					0.261	0.141	0.082	0.111	1.1	0.5	0.3	0.4
	M					0.183	0.120	0.077	0.105	1.2	0.7	0.5	0.6
	L					0.219	0.167	0.134	0.171	2.7	2.0	1.6	1.9
	XL					0.099	0.078	0.038	0.040	1.4	1.1	0.5	0.5
H/L	S	1.5	0.7	0.4	0.6	0.078	0.042	0.027	0.046	0.3	0.2	0.1	0.2
	M	1.5	1.0	0.8	1.1	0.027	0.022	0.022	0.035	0.2	0.1	0.1	0.2
	L	3.9	2.7	2.0	2.4	0.099	0.078	0.063	0.087	1.2	0.9	0.7	1.0
	XL	1.1	0.8	0.5	0.5	0.014	0.012	0.007	0.010	0.2	0.2	0.1	0.1
Non-H/L	S	0.9	0.5	0.3	0.3	0.183	0.098	0.055	0.065	0.7	0.4	0.2	0.2
	M	1.1	0.7	0.4	0.5	0.156	0.098	0.056	0.070	1.0	0.6	0.3	0.4
	L	2.2	1.6	1.3	1.6	0.120	0.089	0.070	0.084	1.5	1.1	0.8	1.0
	XL	1.5	1.2	0.5	0.5	0.085	0.066	0.031	0.030	1.2	0.9	0.4	0.4
White Alone	S	1.0		0.3	0.4	0.150		0.048	0.064	0.6		0.2	0.2
	M	1.2		0.4	0.5	0.131		0.047	0.061	0.8		0.3	0.4
	L	2.4		1.3	1.6	0.106		0.059	0.073	1.3		0.7	0.8
	XL	1.0		0.3	0.3	0.045		0.014	0.014	0.6		0.2	0.2
Black/AA Alone	S	1.0		0.3	0.3	0.037		0.012	0.013	0.2		0.0	0.0
	M	0.8		0.3	0.4	0.022		0.010	0.012	0.1		0.1	0.1
	L	3.2		2.1	2.6	0.059		0.041	0.051	0.7		0.5	0.6
	XL	2.8		1.1	1.1	0.043		0.019	0.019	0.6		0.3	0.3
Asian Alone	S	1.4		0.2	0.4	0.028		0.006	0.011	0.1		0.0	0.0
	M	1.1		0.4	0.6	0.008		0.004	0.007	0.0		0.0	0.0

	L	1.1	0.6	0.7	0.004	0.003	0.005	0.1	0.0	0.1
	XL	0.9	0.2	0.2	0.003	0.001	0.001	0.0	0.0	0.0
AI/AN Alone	S	0.9	0.3	0.4	0.002	0.001	0.001	0.0	0.0	0.0
	M	1.4	0.8	0.8	0.002	0.001	0.001	0.0	0.0	0.0
	L	3.3	1.9	1.7	0.001	0.001	0.001	0.0	0.0	0.0
	XL	1.2	0.6	0.8	0.000	0.000	0.000	0.0	0.0	0.0
NH/PI Alone	S	1.4	0.6	0.5	0.001	0.000	0.000	0.0	0.0	0.0
	M	2.0	1.0	1.2	0.001	0.001	0.001	0.0	0.0	0.0
	L	6.4	2.3	1.7	0.001	0.000	0.000	0.0	0.0	0.0
	XL	0.8	0.3	0.0	0.000	0.000	0.000	0.0	0.0	0.0
Other Alone	S	1.4	0.4	0.5	0.034	0.011	0.013	0.1	0.0	0.0
	M	1.6	0.9	1.4	0.014	0.010	0.013	0.1	0.1	0.1
	L	3.7	2.2	3.0	0.036	0.024	0.031	0.5	0.3	0.4
	XL	0.9	0.5	0.6	0.006	0.004	0.003	0.1	0.1	0.0
2+	S	1.0	0.3	0.4	0.009	0.003	0.004	0.0	0.0	0.0
	M	1.4	0.6	0.9	0.006	0.003	0.005	0.0	0.0	0.0
	L	3.3	1.8	1.9	0.011	0.006	0.005	0.1	0.1	0.1
	XL	1.1	0.4	0.5	0.002	0.001	0.001	0.0	0.0	0.0

c)

	Year	Normalized by Tract H/L or non-H/L Population[a] (%)				Counts (millions)				Normalized by Tract Total Population[b] (%)			
		2000	2005	2010	2015	2000	2005	2010	2015	2000	2005	2010	2015
Population	Group												
Total	S					0.411	0.296	0.195	0.264	5.0	3.5	2.2	2.8
	M					1.672	0.957	0.670	0.865	9.2	5.1	3.4	4.2
	L					1.997	1.541	1.158	1.541	9.9	7.3	5.3	6.8
	XL					1.063	0.843	0.641	0.784	12.4	9.5	7.3	8.7
H/L	S	6.3	4.6	3.2	5.2	0.046	0.040	0.036	0.067	0.6	0.5	0.4	0.7
	M	11.0	6.1	4.1	4.9	0.499	0.316	0.233	0.323	2.7	1.7	1.2	1.6
	L	14.5	10.1	7.6	9.9	0.499	0.425	0.371	0.567	2.5	2.0	1.7	2.5
	XL	13.1	10.1	8.8	11.2	0.292	0.260	0.235	0.346	3.4	2.9	2.7	3.8
Non-H/L	S	4.9	3.3	2.0	2.4	0.365	0.256	0.160	0.197	4.4	3.0	1.8	2.1
	M	8.6	4.7	3.1	3.8	1.173	0.641	0.437	0.542	6.5	3.4	2.2	2.6
	L	9.0	6.6	4.7	5.7	1.498	1.117	0.787	0.973	7.4	5.3	3.6	4.3
	XL	12.1	9.3	6.6	7.3	0.771	0.583	0.406	0.438	9.0	6.6	4.6	4.8
White Alone	S	5.2		2.2	2.7	0.302		0.130	0.166	3.7		1.5	1.8
	M	8.2		3.0	3.8	0.867		0.337	0.445	4.8		1.7	2.2
	L	9.6		4.9	6.3	1.254		0.653	0.877	6.2		3.0	3.9
	XL	10.9		5.5	6.6	0.537		0.266	0.336	6.3		3.0	3.8
Black/AA Alone	S	4.2		1.8	1.8	0.048		0.023	0.025	0.6		0.3	0.3
	M	10.0		2.9	3.2	0.297		0.092	0.099	1.6		0.5	0.5
	L	9.8		6.0	7.2	0.412		0.272	0.332	2.0		1.2	1.5
	XL	15.8		10.7	11.7	0.289		0.209	0.227	3.4		2.4	2.6
Asian Alone	S	3.7		2.0	2.9	0.023		0.016	0.026	0.3		0.2	0.3
	M	12.3		5.4	6.5	0.196		0.113	0.151	1.1		0.6	0.7
	L	9.1		5.1	6.5	0.076		0.065	0.093	0.4		0.3	0.4

	XL	10.3	4.3	5.6	0.044	0.024	0.034	0.5	0.3	0.4
AI/AN Alone	S	4.5	2.3	4.2	0.002	0.001	0.002	0.0	0.0	0.0
	M	8.7	3.1	3.8	0.012	0.005	0.005	0.1	0.0	0.0
	L	9.5	5.0	5.6	0.013	0.009	0.008	0.1	0.0	0.0
	XL	11.9	7.4	10.4	0.004	0.003	0.003	0.1	0.0	0.0
NH/PI Alone	S	4.9	2.7	3.5	0.001	0.001	0.001	0.0	0.0	0.0
	M	8.9	5.8	7.2	0.005	0.004	0.005	0.0	0.0	0.0
	L	14.5	9.2	8.7	0.005	0.004	0.004	0.0	0.0	0.0
	XL	19.2	11.7	11.1	0.002	0.001	0.001	0.0	0.0	0.0
Other Alone	S	6.1	3.3	3.9	0.021	0.016	0.016	0.3	0.2	0.2
	M	10.6	4.1	4.5	0.221	0.090	0.094	1.2	0.5	0.5
	L	12.5	6.3	9.5	0.166	0.111	0.144	0.8	0.5	0.6
	XL	13.9	10.1	14.6	0.157	0.119	0.142	1.8	1.4	1.6
2+	S	5.2	2.4	5.9	0.013	0.008	0.022	0.2	0.1	0.2
	M	10.1	3.5	4.1	0.076	0.028	0.033	0.4	0.1	0.2
	L	12.0	6.1	6.9	0.071	0.043	0.048	0.4	0.2	0.2
	XL	12.5	8.0	8.8	0.030	0.020	0.019	0.3	0.2	0.2

Note: Lacking 2005 race data due to inconsistent reapportionment of "Other Alone".

[a] Normalized by Tract Sub-population: denominator is the subgroup population (e.g., # White alone exposed in tracts / # White alone living in tracts around airports)

[b] Normalized by Tract Total Population: denominator is the total population (e.g., # White alone exposed in tracts / # total population living in tracts around airports)

Abbreviations: AA, African American; AI/AN, American Indian/Alaska Native; dB(A), A-weighted decibels; DNL, day-night average sound level; H/L, Hispanic/Latino; L, large; M, medium; NH/PI, Native Hawaiian/Pacific Islander; S, small; XL, extra-large; 2+, Two or more races.

Supplemental Figure 1. Temporal Trends in Mean Aircraft Noise Contour Size and Airport Enplanements/LTO Operations for 90 U.S. Airports.

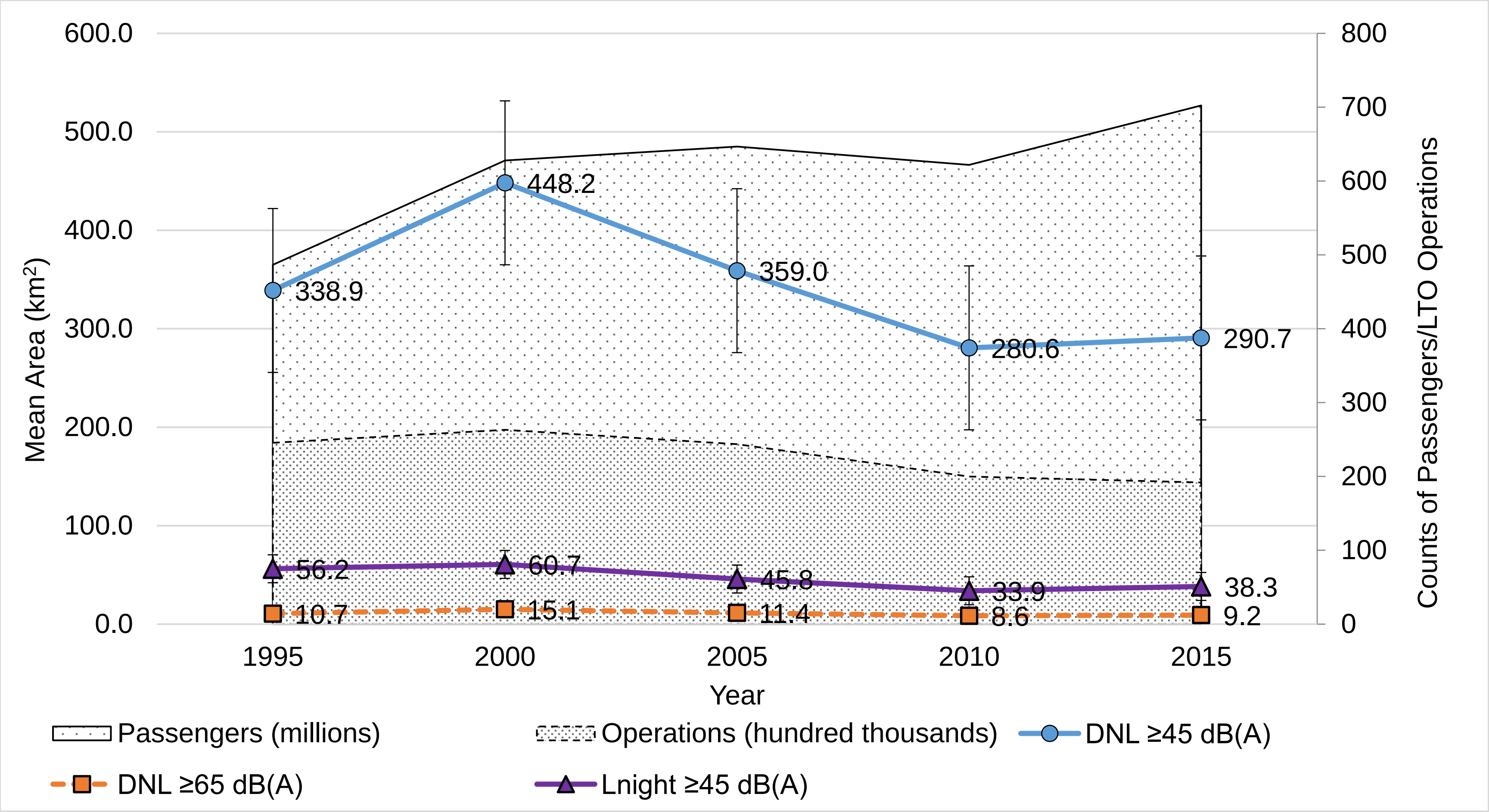

Abbreviations: dB(A), A-weighted decibels; DNL, day-night average sound level; L_{night}, nighttime equivalent sound level; LTO, landing and take-off.

Supplemental Figure 2. Temporal Trends in Noise Contour Areas Across Group-based Trajectories for a) DNL ≥45, b) DNL ≥65, and c) L_{night} ≥45 dB(A).

a)

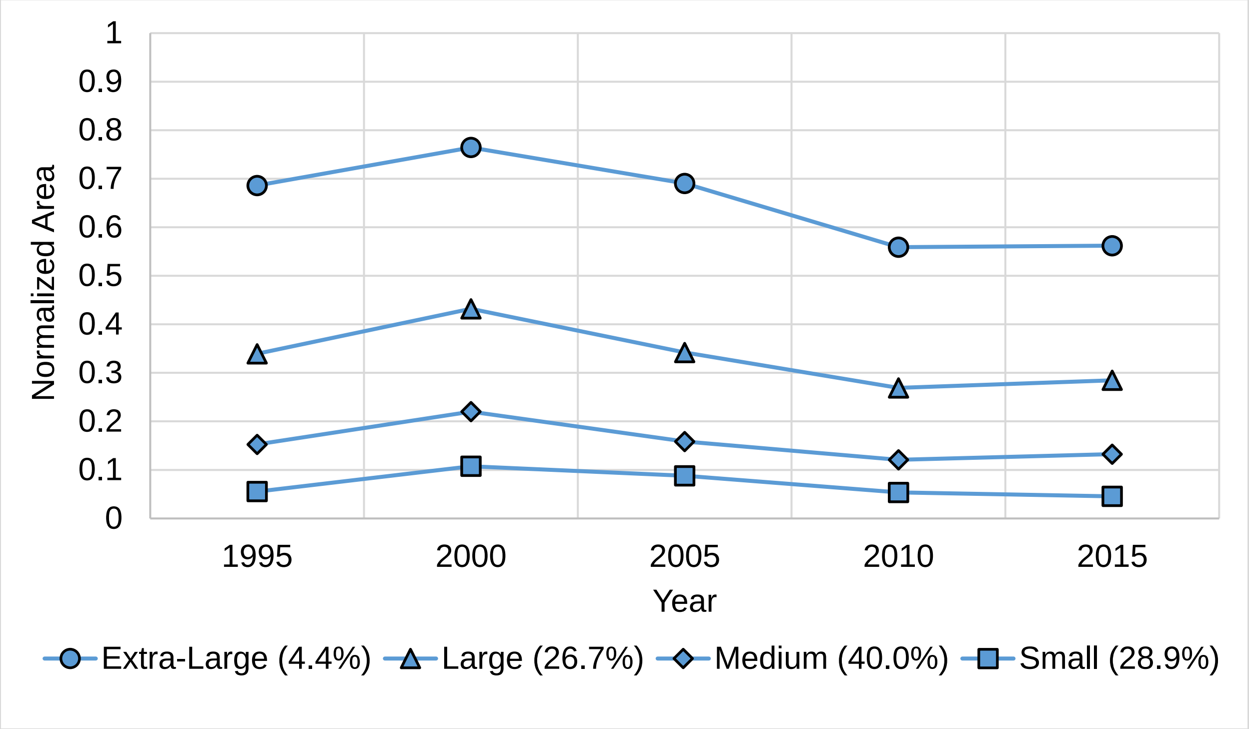

b)

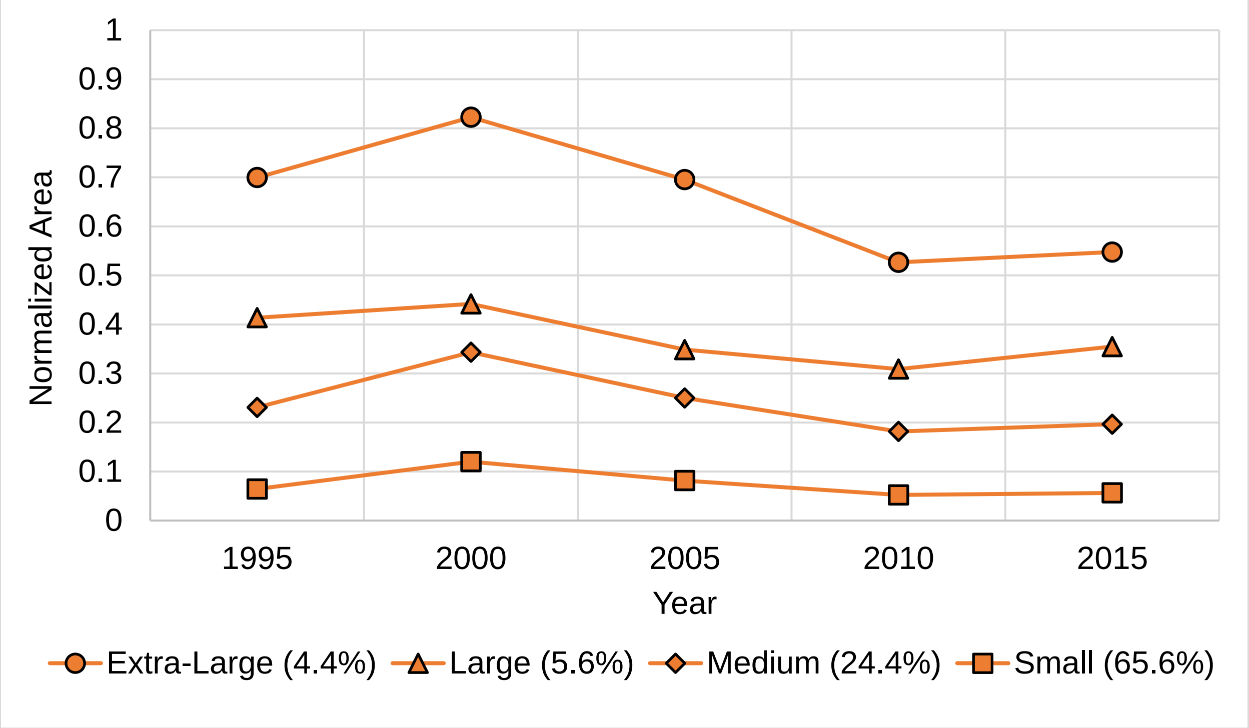

c)

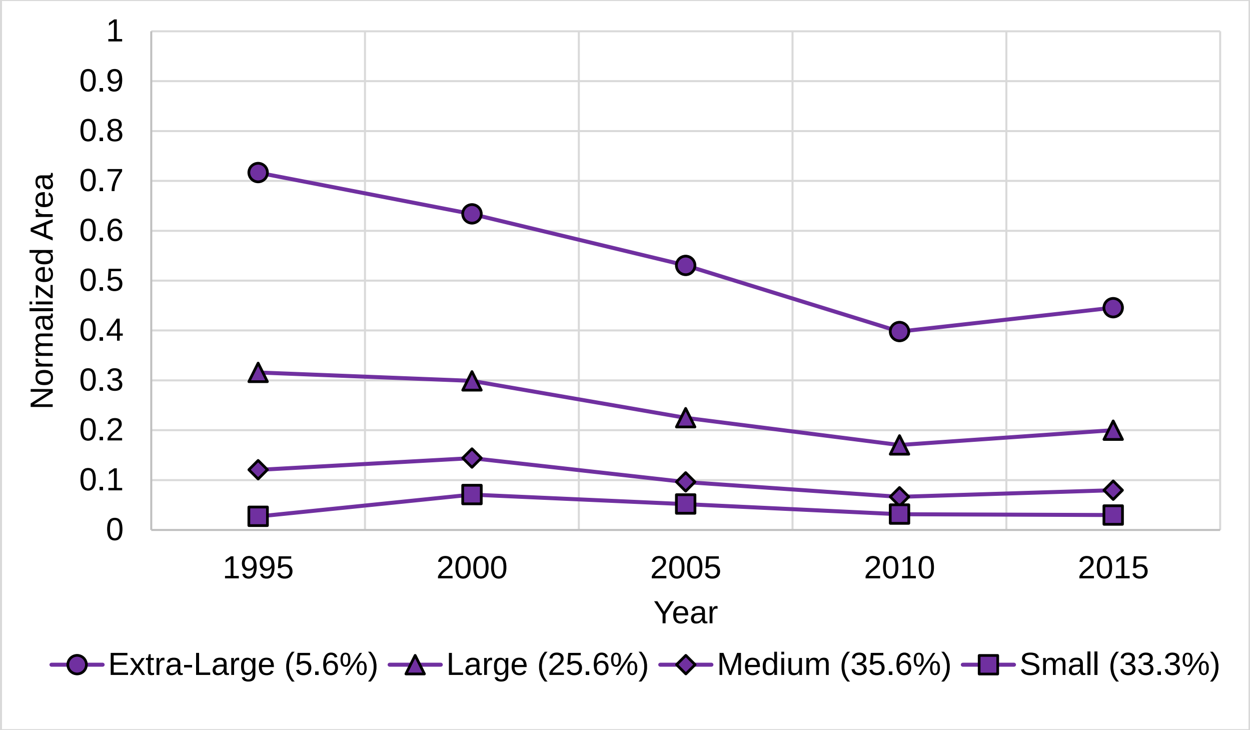

Abbreviations: dB(A), A-weighted decibels; DNL, day-night average sound level; L_{night}, nighttime equivalent sound level.

Supplemental Figure 3. Temporal Trends in Residents Exposed to DNL ≥65 dB(A) Around 90 U.S. Airports by Race, Presented as: a) Normalized by Tract Race Group Population[a], b) Total Counts, and c) Normalized by Tract Total Population[b].

a)

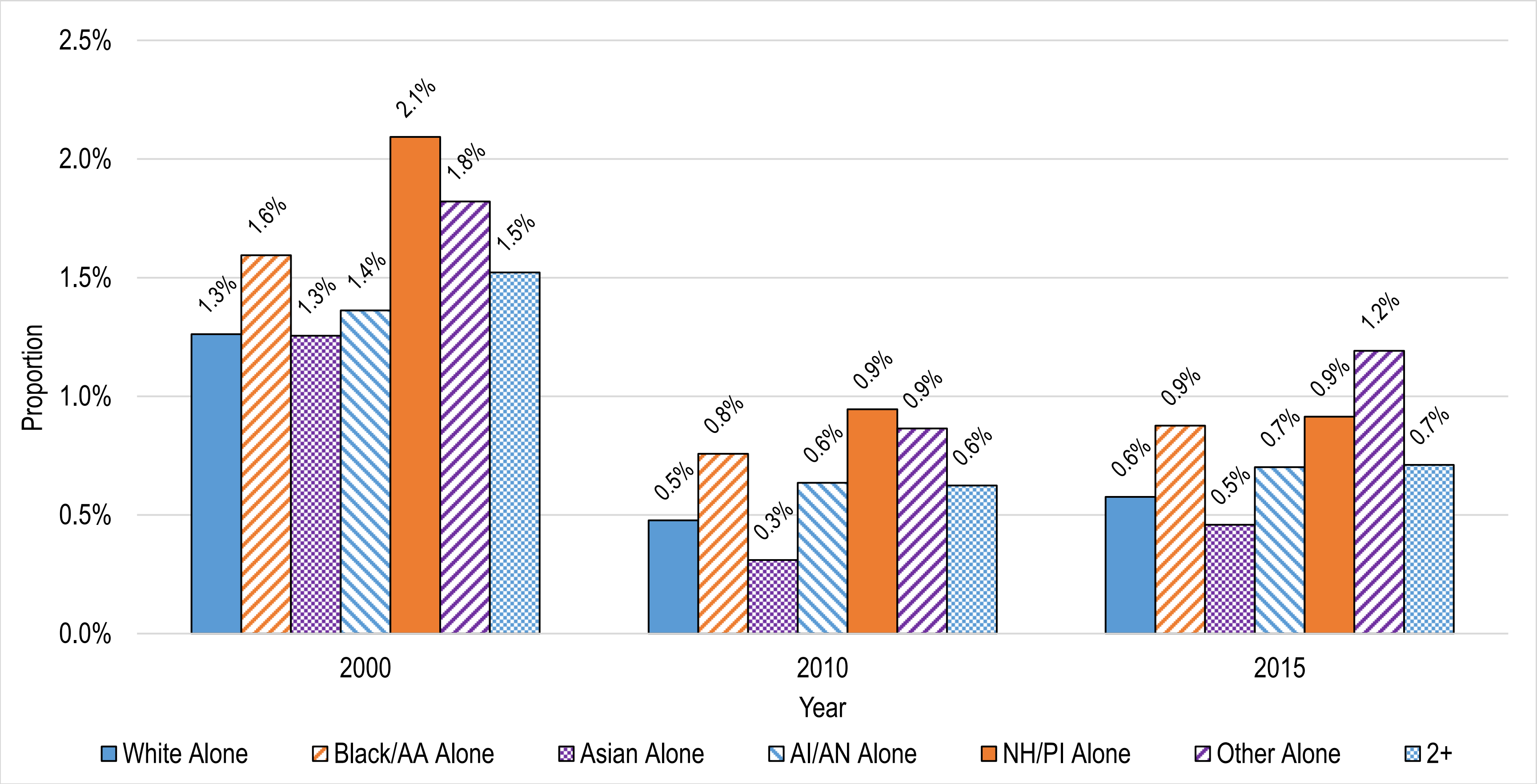

b)

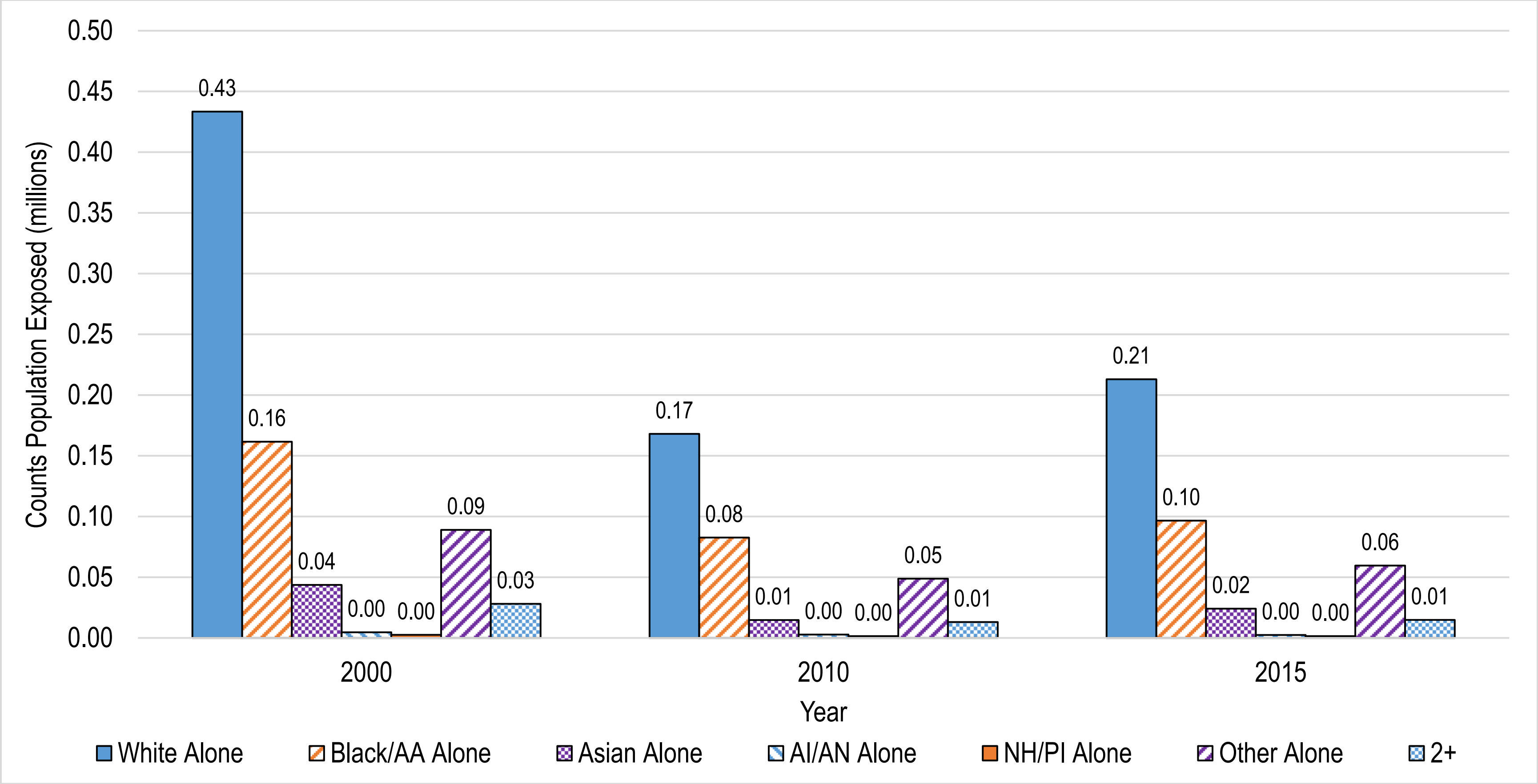
Counts Population Exposed (millions)
0.50
0.45
0.40
0.35
0.30
0.25
0.20
0.15
0.10
0.05
0.00
0.43
0.16
0.04
0.00
0.00
0.09
0.03
0.17
0.08
0.01
0.00
0.00
0.05
0.01
0.21
0.10
0.02
0.00
0.00
0.06
0.01
2000
2010
2015
Year
White Alone
Black/AA Alone
Asian Alone
AI/AN Alone
NH/PI Alone
Other Alone
2+

c)

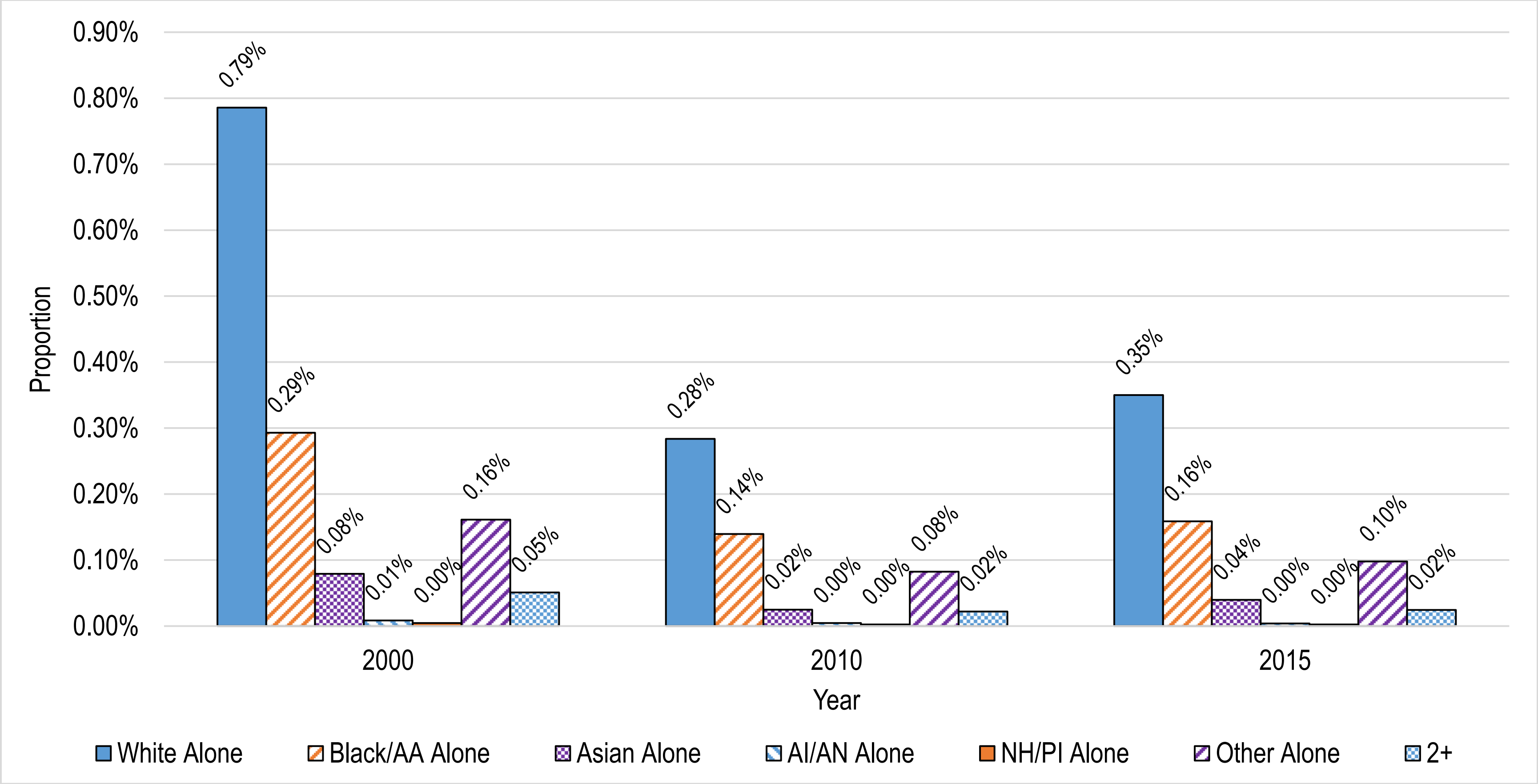

Note: Lacking 2005 race data due to inconsistent reapportionment of “Other Alone”.

[a] Normalized by Tract Sub-population: denominator is the subgroup population (e.g., # White alone exposed in tracts / # White alone living in tracts around airports)

[b] Normalized by Tract Total Population: denominator is the total population (e.g., # White alone exposed in tracts / # total population living in tracts around airports)

Abbreviations: AA, African American; AI/AN, American Indian/Alaska Native; dB(A), A-weighted decibels; DNL, day-night average sound level; H/L, Hispanic/Latino; L, large; M, medium; NH/PI, Native Hawaiian/Pacific Islander; S, small; XL, extra-large; 2+, Two or more races.

Supplemental Figure 4. Temporal Trends in Residents Exposed to L_{night} ≥45 dB(A) Around 90 U.S. Airports by Race, Presented as: a) Normalized by Tract Race Group Population[a], b) Total Counts, and c) Normalized by Tract Total Population[b].

a)

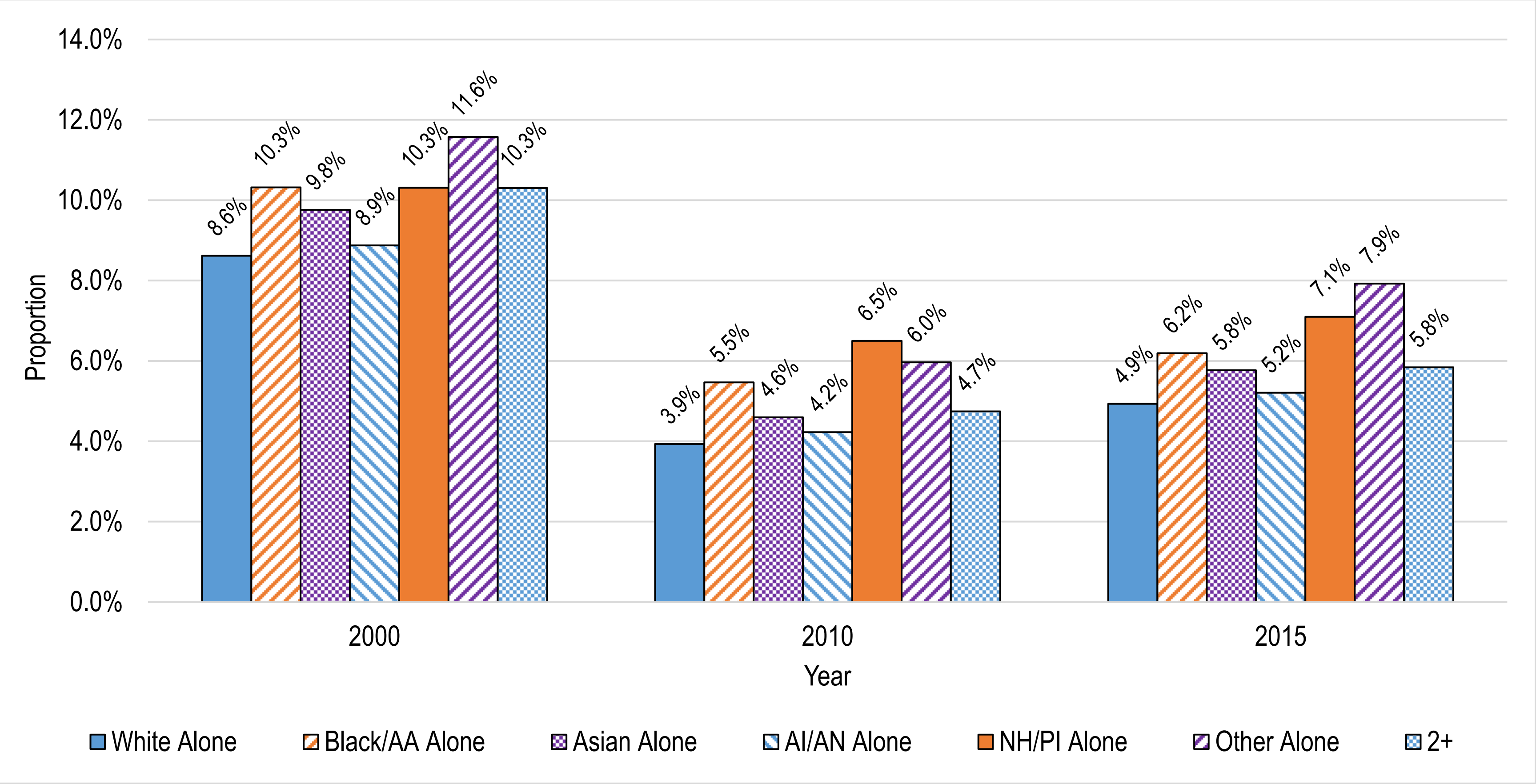

b)

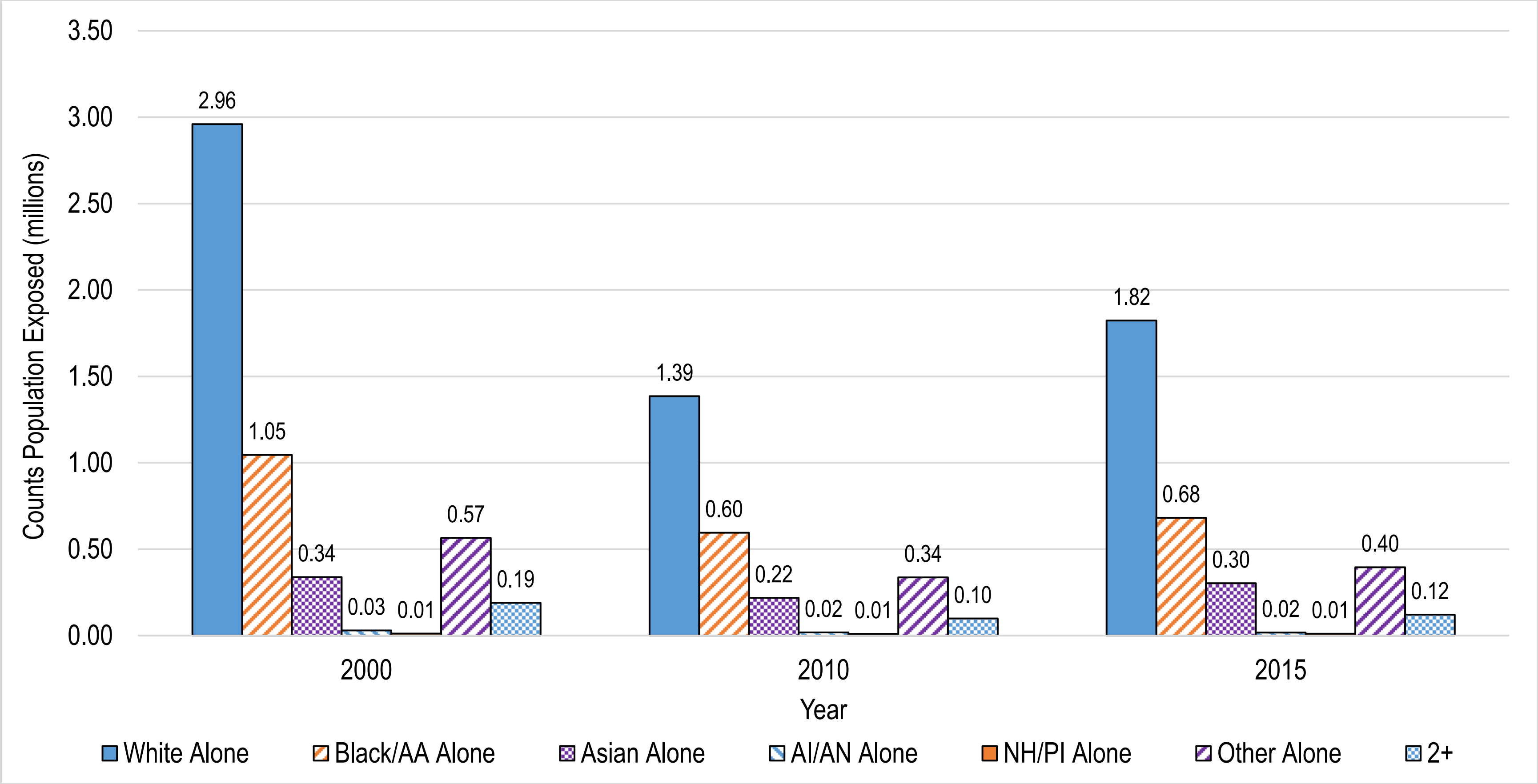

3.50
3.00
2.50
2.00
1.50
1.00
0.50
0.00
Counts Population Exposed (millions)
2.96
1.05
0.34
0.03
0.01
0.57
0.19
1.39
0.60
0.22
0.02
0.01
0.34
0.10
1.82
0.68
0.30
0.02
0.01
0.40
0.12
2000
2010
2015
Year
White Alone
Black/AA Alone
Asian Alone
AI/AN Alone
NH/PI Alone
Other Alone
2+

c)

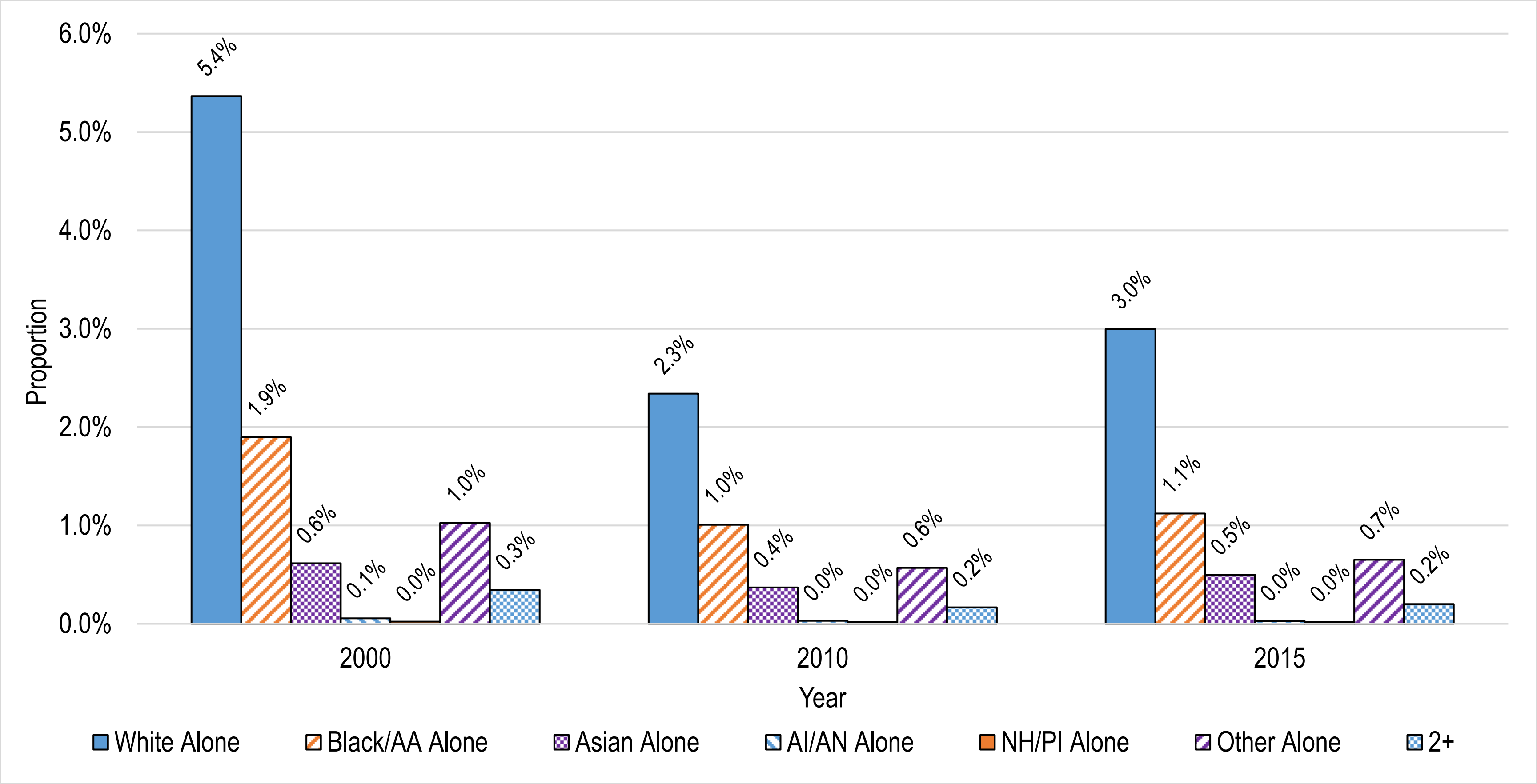

Note: Lacking 2005 race data due to inconsistent reapportionment of "Other Alone".

[a] Normalized by Tract Sub-population: denominator is the subgroup population (e.g., # White alone exposed in tracts / # White alone living in tracts around airports)

[b] Normalized by Tract Total Population: denominator is the total population (e.g., # White alone exposed in tracts / # total population living in tracts around airports)

Abbreviations: AA, African American; AI/AN, American Indian/Alaska Native; dB(A), A-weighted decibels; DNL, day-night average sound level; H/L, Hispanic/Latino; L, large; M, medium; NH/PI, Native Hawaiian/Pacific Islander; S, small; XL, extra-large; 2+, Two or more races.
